# Characterization of *Vitis vinifera *NPR1 homologs involved in the regulation of *Pathogenesis-Related *gene expression

**DOI:** 10.1186/1471-2229-9-54

**Published:** 2009-05-11

**Authors:** Gaëlle Le Henanff, Thierry Heitz, Pere Mestre, Jerôme Mutterer, Bernard Walter, Julie Chong

**Affiliations:** 1 Laboratoire Vigne, Biotechnologies et Environnement (LVBE, EA3991), Université de Haute Alsace, 33 rue de Herrlisheim, 68000 Colmar, France; 2 Département Réseaux Métaboliques chez les Végétaux, IBMP du CNRS (UPR2357), 12 rue du général Zimmer, 67000 Strasbourg, France; 3 Laboratoire de Génétique et Amélioration de la Vigne, INRA et Université de Strasbourg (UMR1131), 28 rue de Herrlisheim, 68000 Colmar, France

## Abstract

**Background:**

Grapevine protection against diseases needs alternative strategies to the use of phytochemicals, implying a thorough knowledge of innate defense mechanisms. However, signalling pathways and regulatory elements leading to induction of defense responses have yet to be characterized in this species. In order to study defense response signalling to pathogens in *Vitis vinifera*, we took advantage of its recently completed genome sequence to characterize two putative orthologs of *NPR1*, a key player in salicylic acid (SA)-mediated resistance to biotrophic pathogens in *Arabidopsis thaliana*.

**Results:**

Two cDNAs named *VvNPR1.1 *and *VvNPR1.2 *were isolated from *Vitis vinifera *cv Chardonnay, encoding proteins showing 55% and 40% identity to Arabidopsis NPR1 respectively. Constitutive expression of *VvNPR1.1 *and *VvNPR1.2 *monitored in leaves of *V. vinifera *cv Chardonnay was found to be enhanced by treatment with benzothiadiazole, a SA analog. In contrast, *VvNPR1.1 *and *VvNPR1.2 *transcript levels were not affected during infection of resistant *Vitis riparia *or susceptible *V. vinifera *with *Plasmopara viticola*, the causal agent of downy mildew, suggesting regulation of VvNPR1 activity at the protein level. VvNPR1.1-GFP and VvNPR1.2-GFP fusion proteins were transiently expressed by agroinfiltration in *Nicotiana benthamiana *leaves, where they localized predominantly to the nucleus. In this system, *VvNPR1.1 *and *VvNPR1.2 *expression was sufficient to trigger the accumulation of acidic SA-dependent Pathogenesis-Related proteins PR1 and PR2, but not of basic chitinases (PR3) in the absence of pathogen infection. Interestingly, when *VvNPR1.1 *or *AtNPR1 *were transiently overexpressed in *Vitis vinifera *leaves, the induction of grapevine *PR1 *was significantly enhanced in response to *P. viticola*.

**Conclusion:**

In conclusion, our data identified grapevine homologs of NPR1, and their functional analysis showed that VvNPR1.1 and VvNPR1.2 likely control the expression of SA-dependent defense genes. Overexpression of *VvNPR1 *has thus the potential to enhance grapevine defensive capabilities upon fungal infection. As a consequence, manipulating *VvNPR1 *and other signalling elements could open ways to strengthen disease resistance mechanisms in this crop species.

## Background

Grapevine (*Vitis vinifera*) is a major fruit crop worldwide that is susceptible to many microbial infections, especially by fungi, thus requiring an intensive use of phytochemicals. The economic costs and negative environmental impact associated with these applications led to search for alternative strategies involving activation of the plant's innate defense system. In order to efficiently limit the losses due to diseases, it is therefore necessary to have a thorough knowledge of grapevine disease resistance mechanisms.

Plants have developed a two-layered innate immune system for defense against pathogens. Primary innate immunity, the first line of defense of plants, is achieved through a set of defined receptors, that recognize conserved microbe-associated molecular patterns [1]. In order to defend themselves against pathogens that can suppress primary defense mechanisms, plants have developed a secondary defense response that is triggered upon recognition of race-specific effectors. Resistance proteins monitor these effectors and subsequently trigger secondary defense responses that often culminate in localized cell death response associated with additional locally induced defense responses, that block further growth of the pathogen [1]. After recognition of the invading microorganism, induced resistance to different types of pathogens is achieved through a network of signal transduction pathways in which the small molecules salicylic acid (SA), jasmonic acid (JA) and ethylene (ET) act as secondary messengers [2]. These regulators then orchestrate the expression of sets of downstream defense genes encoding antimicrobial proteins or enzymes catalyzing the production of defense metabolites. Plant resistance to biotrophic pathogens is classically believed to be mediated through SA signalling [3]. SA accumulation as well as the coordinated expression of *Pathogenesis Related *(*PR*) genes encoding small proteins with antimicrobial activity are also necessary to the onset of Systemic Acquired Resistance (SAR) in plants. SAR is a plant immune response that establishes a broad spectrum resistance in tissues distant from the site of primary infection [4].

In the past years, considerable progress has been made in the model plant *Arabidopsis thaliana *in identifying genes that affect regulation of defense gene expression. Several key plant defense regulators especially involved in the SA signalling pathway have been cloned and characterized [4]. The *npr1 *mutant was isolated in a genetic screen for plants that failed to express *PR2 *gene after SAR induction [5]. NPR1 (Nonexpressor of *PR *genes 1) has been identified as a key positive regulator of the SA-dependent signalling pathway and is required for the transduction of the SA signal to activate *PR *gene expression and Systemic Acquired Resistance [5]. The *NPR1 *gene was cloned in 1997 and shown as encoding a novel protein containing ankyrin repeats involved in protein-protein interactions [6]. *NPR1 *is constitutively expressed and levels of its transcripts increased only two-fold following SA treatment, suggesting that it is regulated at the protein level [7]. Indeed, NPR1 activity is regulated by redox systems which have been recently identified [8]. Inactive NPR1 is present as cytosolic disulfide-bound oligomers in the absence of pathogen attack. Following SA induction, oligomeric NPR1 is reduced to active monomers [9]. NPR1 monomers are translocated to the nucleus where they interact with the TGA class of basic leucine zipper transcription factors, leading to the expression of SA-dependent genes [3,9]. Recent studies have also involved WRKY transcription factors in SA defense responses downstream or in parallel with NPR1 [10].

In Arabidopsis, the NPR1-dependent SA pathway controls the expression of *PR1*, *PR2 *(β-1.3-glucanase) and *PR5 *(thaumatin-like) genes. In contrast, induction of distinct defense genes encoding the defensin PDF1.2 and the PR3 (basic chitinase) proteins is controlled by JA/ET dependent pathways [2].

Originally, the *npr1 *mutant was thought to be only deficient in SA-mediated defense. However, it was shown that NPR1 plays a role in other defense signalling pathways. In *npr1*, the establishment of Induced Systemic Resistance (ISR) in leaves by non-pathogenic root rhizobacteria is blocked. Interestingly, this resistance response is independent of SA but requires ET and JA signalling [11]. Apart from *NPR1*, Arabidopsis genome contains five *NPR1*-related genes called *AtNPR2 *to *AtNPR6 *[12]. Members of the *AtNPR *family encode proteins sharing two domains involved in mediating protein-protein interactions: the Broad Complex, Tramtrack and Bric a brac/Pox virus and Zinc finger (BTB/POZ) domain in the N-terminal and the Ankyrin Repeat Domain (ARD) in the middle of the protein. Whereas AtNPR1 to AtNPR4 have been implicated in signalling of defense responses, AtNPR5 and AtNPR6 (called AtBOP1 and AtBOP2) form a distinct group involved in the regulation of developmental patterning of leaves and flowers [13].

*AtNPR1 *has been over-expressed in Arabidopsis, rice, tomato and wheat, resulting in enhanced bacterial and fungal resistance [7,14-16]. Moreover, homologs of *AtNPR1 *have been cloned and characterized in several crop plants including rice [17], apple [18], banana [19] and cotton [20]. In rice, over-expression of *OsNPR1 *conferred disease resistance to bacterial blight, but also enhanced herbivore susceptibility in transgenic plants [17]. Similarly, over-expression of the *Malus NPR1 *in two apple cultivars resulted in activation of *PR *genes and enhanced resistance to *Erwinia amylovora *and to two important fungal pathogens of apple [18].

In grapevine, many studies described the induction of PR proteins and the production of stilbenes after infection [21,22]. However, signalling pathways and regulatory elements leading to the induction of these responses remain to be characterized in this species. Recently, two genes encoding transcription factors of the WRKY family and potentially involved in grapevine resistance to pathogens have been characterized. Overexpression of *VvWRKY1 *and *VvWRKY2 *in tobacco conferred reduced susceptibility to different types of fungi [23,24].

Recent completion of *Vitis vinifera *genome sequencing in a highly homozygous genotype and in a heterozygous grapevine variety has led to the identification of putative resistance genes and defense signalling elements [25,26]. Based on conserved domain analyses, the grape genome was found to contain a number of genes showing a nucleotide binding site (NBS) and a carboxy-terminal leucine-rich repeat (LRR) typical of resistance (R) genes [26]. Besides putative R genes, the grape genome contains several candidate genes encoding putative signalling components for disease response, with similarity to Arabidopsis EDS1, PAD4, NDR1 and NPR1 [26]. A possible role of the two grapevine regulatory elements sharing sequence similarity to the Arabidopsis SA signalling components NDR1 and EDS1 was recently described by our group [21].

Given the pivotal role of AtNPR1 in plant defense, we decided to take advantage of data from grapevine EST databases and genome sequencing to identify two genes encoding proteins with similarity to AtNPR1, that we called *VvNPR1.1 *and *VvNPR1.2*. Expression of these genes was studied after treatment with benzothiadiazole (BTH, a SA analog) and after inoculation of two resistant or susceptible *Vitis *species with *Plasmopara viticola*, the causal agent of downy mildew. Nuclear localization of VvNPR1.1 and VvNPR1.2 was demonstrated by expressing GFP fusions. To get further insight into VvNPR1 function, the two genes were transiently overexpressed in both *N. benthamiana *and *Vitis vinifera *leaves and consequences on *PR *gene induction were studied.

## Results

### Identification and sequence analysis of two *NPR1*-like genes in *Vitis vinifera*

At the beginning of this study, the grapevine genome was not entirely sequenced. The nucleic acid sequence of *AtNPR1 *(At1g64280) was used to search an EST database of abiotically stressed *Vitis vinifera *cv Chardonnay leaves (EST Analysis Pipeline, ESTAP, [27]). Two ESTs with significant similarity to *AtNPR1 *were identified. Sequence comparison of these two EST with data from grapevine genome sequencing project [28] enabled us to obtain the two full-length cDNAs, named *VvNPR1.1 *(GSVIVT00016536001) and *VvNPR1.2 *(GSVIVT00031933001). Amino acid sequence comparison of VvNPR1.1 and VvNPR1.2 showed that the two proteins display 47% identity and 66% similarity. Completion of *V. vinifera *genome sequencing has revealed only two genes related to "defense" *AtNPRs *(K. Bergeault, unpublished results).

**Figure 1:**
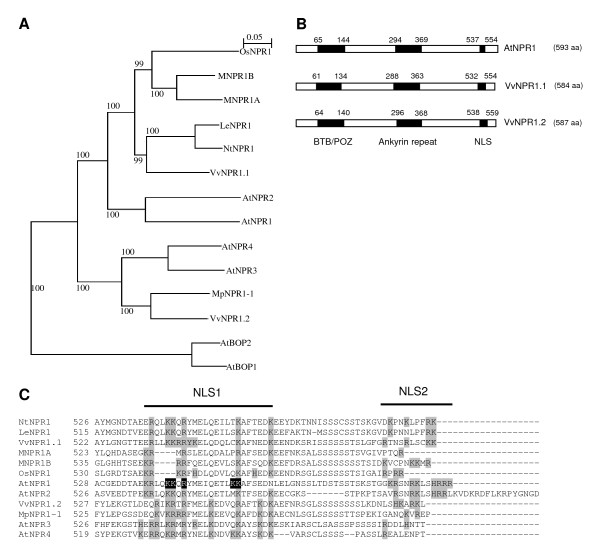
**Comparison of VvNPR1.1 and VvNPR1.2 with other NPR1 homologs and members of *Arabidopsis thaliana *NPR family**. (A) Phylogenetic tree generated with the Phylo_win program using the neighbour-joining method [44]. Sequence alignment was previously realized using the ClustalW tool. Accession numbers are: AtNPR1 (At1g64280), AtNPR2 (At4g26120), AtNPR3 (At5g45110), AtNPR4 (At4g19660), AtBOP1 (At3g57130), AtBOP2 (At2g41370), *Nicotiana tabacum *(NtNPR1, AAM62410.1), *Oryza sativa *cv. japonica (OsNPR1, AAX18700.1), *Lycopersicon esculentum *(LeNPR1, AAT57637.1), *Musa acuminata *(MNPR1A, ABI93182.1; MNPR1B, ABL63913.1), *Malus × domestica *(MpNPR1-1, ACC77697.1) and *Vitis vinifera *(Genoscope accession numbers: VvNPR1.1, GSVIVP00016536001; VvNPR1.2, GSVIVP00031933001). Bootstrap values based on 500 replicates are indicated beside the branches. (B) Schematic representation comparing the structure of AtNPR1, VvNPR1.1 and VvNPR1.2, including the positions of the BTB/POZ domain, the ankyrin repeat domain (ARD) and the nuclear localization signals (NLS). (C) Multiple alignment of putative nuclear localization signals (NLS) at C-terminus of NPRs from different plant species. Basic amino acids are highlighted in grey and residues essential for AtNPR1 nuclear localization [30] are highlighted in black.

Amino acid sequence comparisons showed that VvNPR1.1 has a higher identity with AtNPR1 (55% identity and 75% similarity) than VvNPR1.2 (40% identity and 61% similarity with AtNPR1). VvNPR1.1 and VvNPR1.2 were also compared to NPR1 homologs in different plant species. Phylogenetic analysis (Figure 1A) reveals that VvNPR1.1 groups closely with tobacco and tomato NPR1 proteins (86% and 85% similarity respectively), with NPR1 from monocots and with AtNPR1 and AtNPR2. VvNPR1.2 forms a discrete group with NPR1 from apple (87% similarity), AtNPR3 and AtNPR4.

*VvNPR1.1 *and *VvNPR1.2 *encode putative proteins of 584 and 587 amino acids respectively (Figure 1B). According to PROSITE tool [29], VvNPR1.1 and VvNPR1.2 are predicted to have the same overall organization as members of the AtNPR family, with an amino terminal BTB/POZ domain and a central ankyrin repeat domain (Figure 1B). In addition, the carboxy terminal domains of VvNPR1.1 and VvNPR1.2 are rich in basic amino acids typical of nuclear localization signals (NLS, Figure 1C). Kinkema *et al*. [30] showed that five residues in the C-terminus of AtNPR1 are essential for its nuclear translocation and constitute the NLS1. Four of these five amino acids are conserved in VvNPR1.1 (Figure 1C), whereas some lysine residues have turned into arginine in VvNPR1.2. Basic amino acids of the second NLS in AtNPR1 have been shown to be not necessary for nuclear targeting [30] and are less conserved among the different homologs even in the two grapevine proteins (Figure 1C).

### *VvNPR1.1 *and *VvNPR1.2 *expression following BTH treatment in grapevine leaves

**Figure 2:**
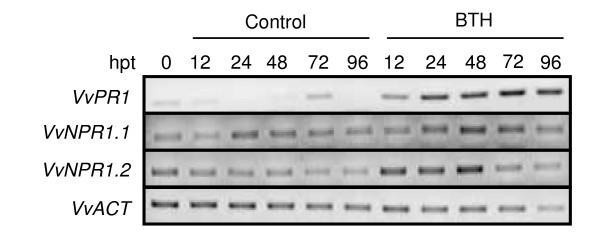
**Expression patterns of *VvNPR1.1 *and *VvNPR1.2 *upon BTH treatment**. Detached leaves of *Vitis vinifera *cv Chardonnay were sprayed with a solution of BTH (80 mg.L^-1^) or water as control. Samples were collected at different time points. Hpt: hours post treatment; 0: untreated leaves at the beginning of the experiment. Actin (*VvACT*) was used as an internal control. Primer sequences are listed in table 1.

In Arabidopsis, *AtNPR1 *is constitutively expressed and can be further stimulated by SA or 2.6-dichloroisonicotinic acid (INA) treatment and by infection with *Hyaloperonospora parasitica *[31]. In order to study the expression profile of the two grapevine *NPR1 *genes, detached leaves of *Vitis vinifera *cv Chardonnay were treated with a solution of BTH (a SA analog). We also monitored the expression of a grapevine *PR1 *gene, a SAR marker, whose sequence is the most closely related to Arabidopsis SA-dependent *PR1 *(GSVIVT 00038575001,[28]). As shown in Figure 2, *VvPR1 *expression was strongly stimulated by BTH as soon as 12 h posttreatment compared to water-treated leaves where *VvPR1 *expression was almost undetectable. *VvNPR1.1 *was constitutively expressed in water-treated leaves, but expression was only slightly upregulated by BTH treatment (Figure 2). Interestingly, *VvNPR1.2*, whose expression was also detectable in control leaves, was further induced by BTH and peaked between 12 to 48 h after treatment (Figure 2). These results show that, as observed in Arabidopsis, *VvNPR1.1 *and *VvNPR1.2 *are constitutively expressed in grapevine and that *VvNPR1.2 *expression can be further enhanced by a SAR inducer.

### Expression patterns of *VvNPR1.1 *and *VvNPR1.2 *during compatible and incompatible interactions with Plasmopara viticola

We have next investigated whether the expression of *VvNPR1.1 *and *VvNPR1.2 *could be modulated after pathogen infection and whether their expression was differentially affected during compatible or incompatible interactions. Grapevine and related species exhibit a wide spectrum of resistance to the biotrophic pathogen *Plasmopara viticola*, the downy mildew agent. Two different *Vitis *species, the resistant *Vitis riparia *cv Gloire de Montpellier and the susceptible *Vitis vinifera *cv Chardonnay, were challenged with *Plasmopara viticola *or water as control. The expression patterns of *VvNPR1.1 *and *VvNPR1.2 *were determined after inoculation using real-time quantitative PCR. The expression of each gene after inoculation was calculated as fold induction compared to H_2_O-inoculated leaves at the same time point as described by Pfaffl *et al *[32].

**Figure 3:**
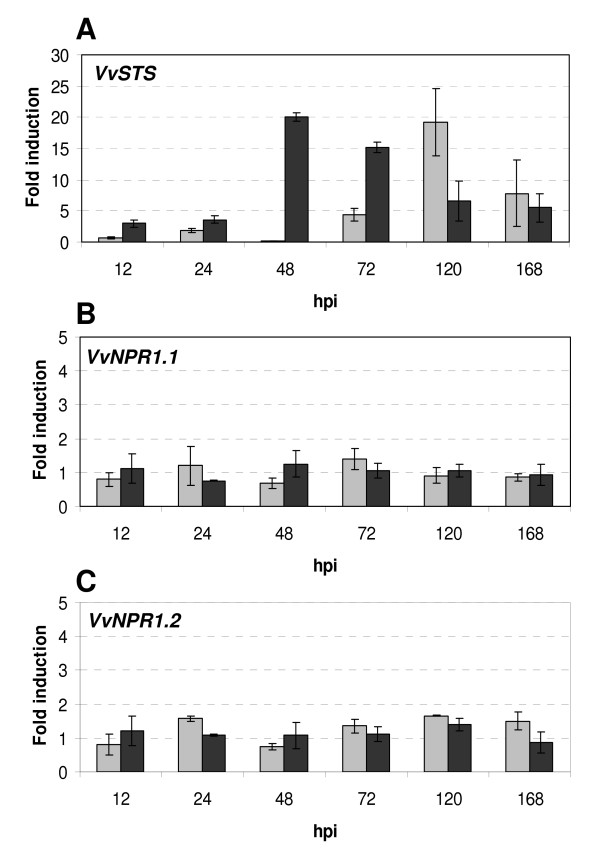
**Expression patterns of *VvNPR1.1 *and *VvNPR1.2 *during a compatible or an incompatible interaction between grapevine and *Plasmopara viticola***. Leaves of plantlets of *Vitis vinifera *cv Chardonnay (grey bars) and *Vitis riparia *cv Gloire de Montpellier (dark bars) were inoculated with *Plasmopara viticola *(1.5 × 10^5 ^spores mL^-1^). Control leaves were sprayed with water. Leaves were collected at different time points as indicated. Hpi: Hours post inoculation. Transcript levels of each gene (Stilbene synthase *VvSTS *(A); *VvNPR1.1 *(B); *VvNPR1.2 *(C)) were normalized to actin transcript levels. The fold induction indicates normalized expression levels in inoculated leaves compared to normalized expression levels observed in water-treated leaves at the same time point. Expression ratio at the beginning of the experiment (0) is set to 1. Mean values and standard deviations were obtained from 2 duplicate experiments.

Five days after inoculation with *P. viticola*, a number of necrotic spots were observed on leaves of the resistant species *V. riparia*, whereas sporangia covered almost the entire leaf surface of the susceptible *V. vinifera *(data not shown). Expression of a stilbene synthase gene (*VvSTS*) was determined as a positive control of defense gene induction by *P. viticola *infection. As expected,*P. viticola *inoculation triggered *VvSTS *expression in both susceptible and tolerant *Vitis *species (Figure 3A). However, *VvSTS *expression was enhanced much earlier in resistant *V. riparia*, where transcripts began to accumulate 12 h after inoculation and were stimulated about 20-fold at 2 days. In contrast, maximal induction of *VvSTS *expression was measured 5 days after inoculation in *V. vinifera *cv Chardonnay (Figure 3A). Thus, *VvSTS *transcript accumulation was delayed in susceptible *V. vinifera *cv Chardonnay compared to resistant *V. riparia*.

Transcript accumulation of *VvNPR1.1 *and *VvNPR1.2 *was then quantified after *P. viticola *infection. As shown in Figure 3B and 3C, no significant change in the expression of these two genes was detectable for either genotype. Other studies from our group have shown that constitutive expression of *VvNPR1.1 *and *VvNPR1.2 *was also not affected by infection with *Botrytis cinerea *or with *Pseudomonas syringae *pv pisi (data not shown). Taken together, expression studies suggest that *VvNPR1.1 *and *VvNPR1.2 *are not regulated at transcriptional level upon pathogen infection.

### Subcellular localization of VvNPR1.1 and VvNPR1.2

**Figure 4:**
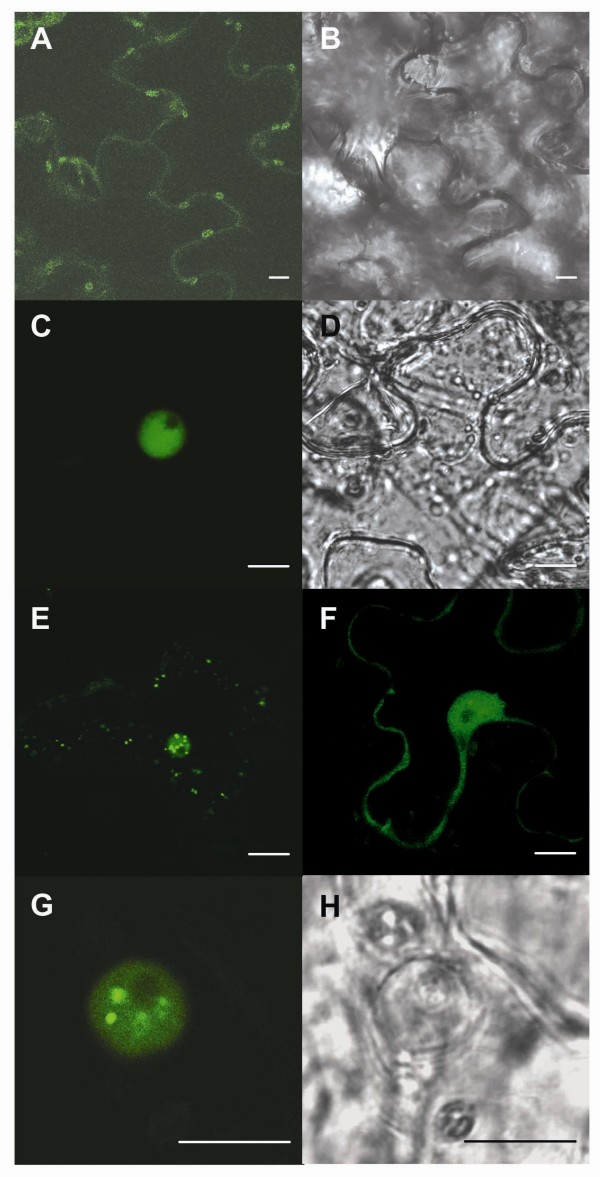
**Subcellular localization of VvNPR1.1 and VvNPR1.2**. *N. benthamiana *leaves were infiltrated with *A. tumefaciens *GV3101 containing empty vector (pK7FWG2) encoding free GFP (A, B), or *AtNPR1 *(C, D), *VvNPR1.1 *(E, G, H), and *VvNPR1.2 *(F) in pK7FWG2. Confocal images were captured 3 days after infiltration. GFP images (A, C, E, F, G) and differential contrast images (B, D, H) of *N. benthamiana *epidermal cells were compared to show the subcellular localization of GFP, AtNPR1-GFP, VvNPR1.1-GFP and VvNPR1.2-GFP. Bar = 10 μM.

The amino acid sequences of both VvNPR1.1 and VvNPR1.2 were found to contain a putative nuclear localization signal (NLS1) in the C terminus of the protein (Figure 1C). To determine the subcellular localization of VvNPR1.1 and VvNPR1.2, the coding regions of *VvNPR1.1*, *VvNPR1.2*, and *AtNPR1 *were fused to 5'-terminus of *eGFP *under the control of the CaMV 35S promoter. The resulting constructs were introduced into *Nicotiana benthamiana *following transient transformation by agroinfiltration. Leaf sectors of agroinfiltrated *N. benthamiana *were observed 3 days after infiltration for GFP fluorescence by confocal microscopy (Figure 4). GFP fluorescence levels were comparable with the 3 constructions studied. Control leaves expressing free GFP yielded a weak fluorescence predominantly visible in the cytoplasm (Figure 4A and 4B). As described previously [30], the AtNPR1-GFP fusion protein fluorescence strongly labelled the nucleus (Figure 4C and 4D). Consistent with the presence of the NLS1, VvNPR1.1-GFP and VvNPR1.2-GFP fusion proteins were localized to the nucleus and to a lesser extent to the cytoplasm both in mesophyll and epidermal cells (Figure 4E and 4F). Localization of GFP fluorescence to nucleus was further observed in cells from peeled epidermis transiently transformed with *VvNPR1.1 *(Figure 4G and 4H). Treatment of *N. benthamiana *leaves with SA 48 h before observation did not influence the localization of the fusion proteins (data not shown).

### Transient expression of *VvNPR1.1 *and *VvNPR1.2 *in *N. benthamiana *triggers the accumulation of acidic PR1 and PR2 but not of PR3

**Figure 5:**
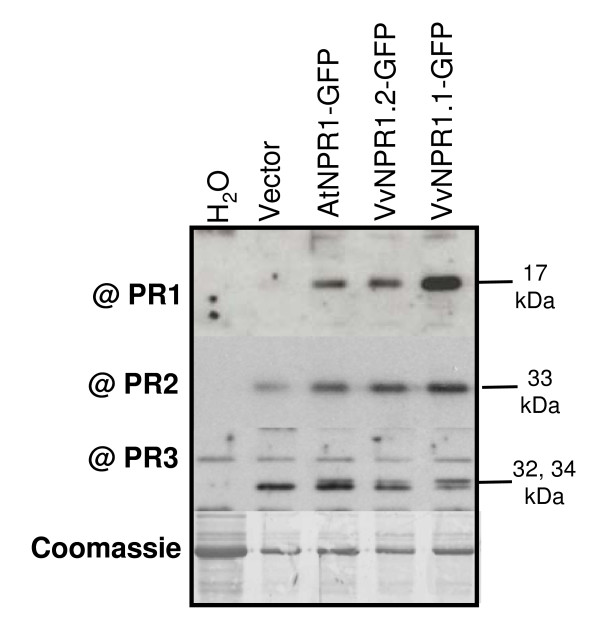
**Induction of PR1 and PR2 accumulation in *N. benthamiana *by transient expression of *VvNPR1.1 *and *VvNPR1.2***. *N. benthamiana *leaves were infiltrated with water (H_2_O) or *A. tumefaciens *GV3101 containing *VvNPR1.1*, *VvNPR1.2*, or *AtNPR1 *in pK7FWG2 or empty vector. Leaves were harvested 3 days after agroinfiltration. Soluble proteins were extracted, submitted to SDS-PAGE and probed with sera against tobacco PR1, PR2 or basic chitinases (PR3).

To investigate if VvNPR1.1 and VvNPR1.2 could control the expression of *PR *genes (especially the *PR1 *gene), PR protein accumulation was analyzed after transient expression of *AtNPR1-GFP*, *VvNPR1.1-GFP *and *VvNPR1.2-GFP*. Leaves of *N. benthamiana *were analyzed 3 days after agroinfiltration for PR protein production by Western blot with anti sera raised against tobacco PR proteins. PR proteins were undetectable in untreated leaves (Figure 5). Transient expression of *AtNPR1-GFP*, *VvNPR1.1-GFP *and *VvNPR1.2-GFP *was sufficient to trigger accumulation of acidic PR1, in contrast to expression of empty vector (encoding free GFP) which produced no signal (Figure 5). In order to determine if another marker of the SA pathway could be enhanced by *VvNPR1 *expression, the same analysis was performed to detect acidic β-1.3 glucanase (PR2). Agroinfiltration of vector alone triggered the expression of PR2 compared to infiltration with H_2_O (Figure 5). However, transient expression of *AtNPR1-GFP*, *VvNPR1.1-GFP *and *VvNPR1.2-GFP *induced a stronger accumulation of PR2 compared to infiltration with empty vector (Figure 5). In order to determine if PR protein induction by AtNPR1 and VvNPR1 is specific of SA signalling, we analyzed the accumulation of basic chitinase (PR3), a SA- independent marker whose expression is controlled by the JA/ET pathway in Arabidopsis [2]. Anti-PR3 serum recognized two proteins of 32 and 34 kDa corresponding to the two basic chitinase isoforms described in tobacco [[33], Fig 5]. Similarly to PR2, agroinfiltration with empty vector triggered the expression of PR3 compared to infiltration with H_2_O (Figure 5). However, in contrast to PR1 and PR2, expression of *AtNPR1-GFP*, *VvNPR1.1-GFP *and *VvNPR1.2-GFP *did not modify significantly PR3 accumulation compared to empty vector (Figure 5).

Similar results concerning PR protein expression were observed after infiltration of *N. benthamiana *with *Agrobacterium *harbouring the coding regions of *AtNPR1*, *VvNPR1.1 *and *VvNPR1.2 *under the control of the 35S CaMV promoter in a pBinplus vector devoid of GFP (data not shown).

### Transient expression of *AtNPR1 *and *VvNPR1.1 *in grapevine leaves enhances accumulation of *VvPR1 *transcripts

**Figure 6:**
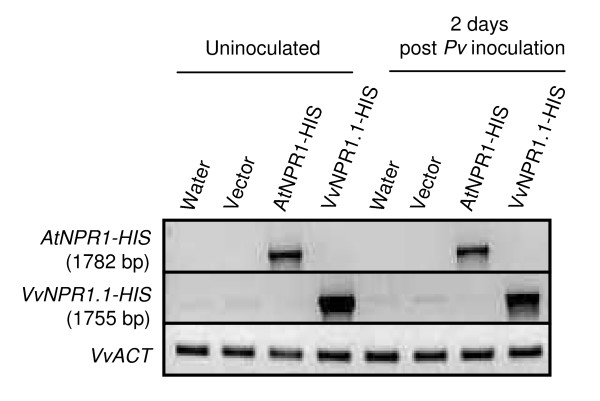
**Detection of *AtNPR1 *and *VvNPR1.1 *transgene expression in grapevine leaves**. Leaves from *in vitro *grown *V. vinifera *cv Syrah were infiltrated with *A. tumefaciens *transformed with pBIN+ carrying *AtNPR1 *or *VvNPR1.1*. Control plants were infiltrated with water. Infiltrated leaves were challenged with *P. viticola *3 days after agroinfiltration. Total RNAs were extracted 3 days after agro-infiltration (uninoculated) and 2 days after *P. viticola *inoculation. Full-lenght mRNA from each transgene was specifically amplified after reverse transcription with primers listed in table 1. *VvACT *was used as internal control.

Heterologous expression in *N. benthamiana *showed that VvNPR1.1 and VvNPR1.2 were able to trigger the accumulation of acidic PR1 and PR2 in the absence of pathogen inoculation. To evaluate the effect of *VvNPR1 *expression in a homologous system (*Vitis vinifera*), we used a recently described protocol of transient gene expression by vacuum agroinfiltration in grapevine [34]. *AtNPR1 *and *VvNPR1.1*, which is the most closely related to *AtNPR1*, were transiently expressed in leaves of *V. vinifera *cv Syrah, a genotype showing high efficiency of transient expression [34]. Gene expression was first analyzed 3 days after agroinfiltration. Grapevine leaves were also later inoculated with *P. viticola *3 days after agroinfiltration and analyzed 2 days after oomycete inoculation. To confirm that *AtNPR1 *and *VvNPR1.1 *were expressed in agroinfiltrated grapevine leaves, we monitored the accumulation of full length transgene-derived mRNAs of *AtNPR1 *and *VvNPR1.1 *by RT-PCR as shown in Figure 6. No PCR amplification was revealed when omitting the reverse transcription step (data not shown).

**Figure 7:**
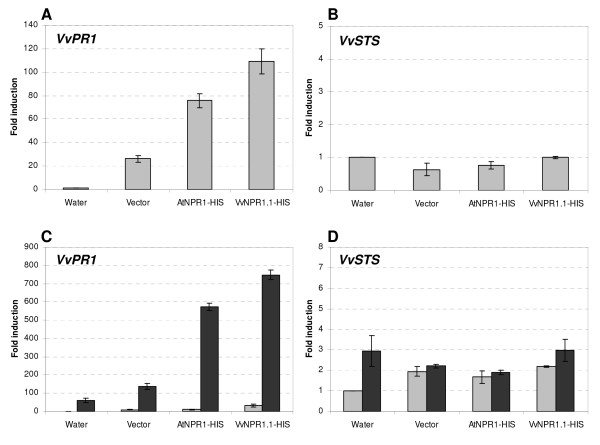
**Expression of *VvPR1 *and *VvSTS *after transient overexpression of *AtNPR1 *and *VvNPR1.1 *in grapevine leaves**. (A, B) Expression levels of *VvPR1 *(A) and *VvSTS *(B), in uninoculated leaves, 3 days after agro-infiltration. (C, D) Expression levels of *VvPR1 *(C) and *VvSTS *(D) in uninoculated and inoculated leaves. Leaves were infiltrated with *Agrobacterium *carrying the different constructs and expression of *VvPR1 *and *VvSTS *was analyzed 3 days later (grey bars). Three days after agroinfiltration, leaves were inoculated with *P. viticola *and expression of genes of interest was analyzed 2 days after inoculation (black bars). Fold induction indicates expression levels in agroinfiltrated leaves compared to the expression in non-inoculated water-infiltrated leaves, which is set to 1. Mean values and standard deviations were obtained from 2 duplicate experiments.

Real time quantitative PCR was used to study the expression of *VvPR1 *and *VvSTS *in grapevine leaves expressing *AtNPR1 *and *VvNPR1.1*, 3 days after agroinfiltration. As shown in Figure 7A, infiltration with empty vector stimulated the expression of *VvPR1*, probably because of the agroinfiltration stress. Interestingly, in leaves expressing *AtNPR1 *and *VvNPR1.1*, a stronger increase in *VvPR1 *transcript accumulation was measured (Figure 7A). In contrast, no significant increase in *VvSTS *transcript accumulation was measured in leaves expressing *AtNPR1 *and *VvNPR1.1 *compared to H_2_O-infiltrated leaves (Figure 7B). In another experiment, we inoculated grapevine leaves with *P. viticola *3 days after agroinfiltration and analyzed gene expression 2 days after inoculation. *VvPR1 *expression was induced by fungal infection as expected. Consistent with the results obtained in uninoculated leaves, *VvPR1 *stimulation in infected leaves was clearly higher in leaves expressing *AtNPR1 *and *VvNPR1.1 *than in leaves preinfiltrated with control *Agrobacterium *(Figure 7C). Although *VvSTS *expression was stimulated 3 fold by infection, no significant effect on its expression was observed when leaves were preinfiltrated with the different constructs (Figure 7D).

Together, these results show that transient expression of both *AtNPR1 *and *VvNPR1.1 *in *Vitis vinifera *is able to enhance expression of a grapevine defense gene known to be controlled by the SA signalling pathway in model plants.

## Discussion

In order to characterize defense response signalling components in grapevine, we identified two homologs of *AtNPR1 *in *Vitis vinifera *cv Chardonnay. Our study provides the first elements for the functional characterization of VvNPR1.

Expression studies of *VvNPR1.1 *and *VvNPR1.2 *showed that these genes are constitutively expressed and that expression can be further enhanced by treatment with BTH, a SA analog. Induction of *NPR1 *genes by treatment with SA or its analogs has been described in a number of plant species including Arabidopsis, mustard, apple, rice, banana and cotton [4,17-20,35]. Interestingly, *VvNPR1.2 *is the most responsive to BTH induction and forms a phylogenetically related group with MpNPR1, AtNPR3 and AtNPR4 which are also highly induced by BTH or INA (another SA analog) respectively [18,36]. In rice, it has been shown that *OsNPR1 *is more rapidly induced in the incompatible interactions leading to resistance than in the compatible interactions leading to disease [17]. Similarly, *MNPR1A *from banana was induced earlier and to higher levels after infection in a *Fusarium oxysporum *tolerant cultivar than in a sensitive one [19]. To evaluate if *VvNPR1 *expression could be differentially regulated during compatible or incompatible interactions between *Vitis *species and *Plasmopara viticola*, we examined the expression of both genes after inoculation of susceptible *Vitis vinifera *cv Chardonnay or resistant *Vitis riparia *cv Gloire de Montpellier with downy mildew. The expression of a gene encoding a stilbene synthase, an enzyme involved in the synthesis of phytoalexins, which is known to be stimulated by *P. viticola *infection was also studied as a positive control. We detected a faster induction of *STS *gene expression after inoculation of the resistant genotype (*Vitis riparia*), consistent with an earlier induction of defense genes in incompatible versus compatible interactions [37]. However, no significant changes in transcript levels were detected for both *VvNPR1.1 *and *VvNPR1.2 *after infection with downy mildew. Overall, the constitutive expression of *VvNPR1 *and the absence of transcriptional regulation after pathogen infection suggest that VvNPR1 activity is regulated at the protein level in grapevine, as previously described in Arabidopsis [4].

In order to address VvNPR1 function, particularly its subcellular localization and its ability to regulate defense gene expression, we first used an heterologous system for transient expression by agroinfiltration of *N. benthamiana *leaves. This method has been described as a rapid and efficient system for the *in vivo *analysis of plant transcription factors and promoters of *PR *genes [38]. The predicted amino acid sequences of VvNPR1.1 and VvNPR1.2 were found to contain a putative nuclear localization signal (NLS1) in their C terminus. Consistently, transiently expressed VvNPR1-GFP and AtNPR1-GFP fusion proteins were localized predominantly to the nucleus, even in the absence of the SAR inducer SA. Constitutive nuclear localization was also revealed by transient expression of AtNPR1-GFP after bombardment of epidermal onion cells [30]. By contrast, in stable transformants, exclusive nuclear localization of AtNPR1-GFP, which is required for activation of *PR *gene expression, was triggered only after treatment with a SAR inducer or infection with a pathogen [30]. Similarly, Arabidopsis lines overexpressing AtNPR1 under the control of the constitutive 35S CaMV promoter and grown under non-inducing conditions have not revealed an increase in the basal level of *PR *genes, indicating that AtNPR1 is essentially inactive in the absence of pathogen infection. *NPR1*-overexpressing plants will thus not activate SA-dependent defense responses until they are challenged with a pathogen [7].

In this study, we showed by transient expression that VvNPR1.1 and VvNPR1.2 are functional in triggering the accumulation of acidic PR1 and PR2 in *N. benthamiana*. This effect was obtained in the absence of an exogenous inducer and correlated with the nuclear localization of VvNPR1.1 and VvNPR1.2. It is likely that agroinfiltration of *N. benthamiana *leaves itself induces a biotic stress that activates responses related to SAR, including targeting of NPR1 proteins to the nucleus. This hypothesis is supported by a higher basal level of PR proteins in empty vector-agroinfiltrated leaves compared to leaves infiltrated with water (Figure 5). Similarly, it has been reported that *Agrobacterium*-mediated transient assays of stress-inducible *PR *promoters have relatively high levels of GUS activity in water and mock-treatments [38]. Finally, it appears that both grapevine NPR1 are active in *N. benthamiana*, in agreement with the ability of AtNPR1 to activate defense responses in other plant species such as rice and wheat [14,16]. Induction of PR protein accumulation was rather specific of defense markers that have been demonstrated to be SA-specific in tobacco [39]. Conversely, *NPR1 *expression had no significant effect on basic chitinase (PR3) accumulation. In Arabidopsis, PR3 represents an SA- independent marker whose expression is controlled by the JA/ET pathway [2]. Moreover, class I basic chitinase expression is activated by overexpression of an ethylene-responsive transcription factor (ERF) in tobacco cells [40].

In order to gain further information on VvNPR1 activity in a homologous system, we used a recently described method of *Agrobacterium*-mediated transient gene expression in *Vitis vinifera *[34]. This system circumvents the time consuming process of generating stable transgenic lines in grapevine. In this study, we provide a first example of successful use of *Agrobacterium*-mediated transient expression for functional analysis of signalling elements in grapevine. *AtNPR1 *and *VvNPR1.1 *were successfully expressed at relatively high level in leaves of *V. vinifera *cv Syrah after agroinfiltration. Transient expression of these two signalling genes resulted in increased *VvPR1 *gene expression in both uninoculated and in *P. viticola *inoculated leaves. In inoculated tissues, the expected stimulation of *PR1 *expression by *P. viticola *was observed; however, *PR1 *expression was further enhanced in infected leaves overexpressing *AtNPR1 *or *VvNPR1.1*. It is likely that the activity of the NPR1 proteins is enhanced by *P. viticola *inoculation. Moreover, it appeared that VvNPR1.1 had a stronger activity than AtNPR1 on induction of *PR1 *expression in grapevine.

Transient expression in *N. benthamiana *and *V. vinifera *shows that VvNPR1.1 and VvNPR1.2 have a positive activity on the expression of *PR1 *and *PR2 *genes (Figure 5). It is thus likely that as in other plant species, VvNPR1 controls the expression of a set of SA-responsive defense genes in grapevine. However, it remains to be determined if VvNPR1.1 and VvNPR1.2 perform different functions in grapevine defense. Arabidopsis genome contains 3 additional genes closely related to *AtNPR1*, which are likely involved in plant defense responses [36], and 2 other more distant genes, *AtBOP1 *(*AtNPR5*) and *AtBOP2 *(*AtNPR6*), with functions in the control of growth asymmetry in leaf and floral patterning [13]. Among NPRs involved in plant defense, phylogenetic analysis revealed that AtNPR1 and AtNPR2 form a subgroup, whereas AtNPR3 and AtNPR4 form a distinct pair [36]. Interestingly, grapevine genome sequencing revealed only two genes related to "defense" *AtNPRs*. VvNPR1.1 belongs to the subgroup comprising AtNPR1 and AtNPR2, and VvNPR1.2 forms a distinct subgroup with AtNPR3, AtNPR4 and MpNPR1-1 from apple (Figure 1). Curiously, a hallmark of this second subgroup is a high inducibility of gene expression by BTH or its analogs [[18,36] and this study]. Different members of the AtNPR family appear to mediate different functions in plant defense. AtNPR1 has been identified as a key positive regulator of SA-dependent gene expression that is required for SAR establishment as well as for basal resistance to virulent pathogens [4]. On the other hand, AtNPR3 and AtNPR4 have been proposed to act as negative regulators of plant defense, since the double *npr3npr4 *mutant shows elevated basal *PR1 *expression and enhanced resistance to virulent bacterial and oomycete pathogens [36]. However, the negative regulation of defense mechanisms by AtNPR3 and AtNPR4 is in contradiction with another study where *npr4 *single mutants were shown to be more susceptible to the virulent bacterial pathogen *Pseudomonas syringae *pv. tomato DC3000 [12]. In this study, AtNPR4 was also implicated in the regulation of JA-inducible genes and in the cross-talk between the SA- and the JA-dependent signalling pathways [12]. Even if VvNPR1.2 is closely related to AtNPR3 and AtNPR4, it is likely not acting as a negative regulator of defense genes since its expression in *N. benthamiana *resulted in enhanced PR1 and PR2 accumulation. Moreover, VvNPR1.2 is closely related to MpNPR1-1, whose overexpression led to activation of *PR *genes and resistance to bacterial and fungal pathogens in apple [18] (Figure 1). Therefore, phylogenetic analysis is not sufficient to predict a positive or negative control of defense responses for a given member of the NPR family. However, it is likely that the two *NPR1 *homologs identified in grapevine do not perform fully overlapping functions. Overexpression or silencing of the two genes in grapevine will help to clarify their respective role in resistance to different pathogens in the future.

## Conclusion

We show here that genome sequence resources combined with transient expression in heterologous and homologous systems allow to obtain rapidly functional information on grapevine genes. The upregulation of acidic *PR1 *and *PR2 *expression by VvNPR1 both in *N. benthamiana *and *Vitis vinifera *strongly suggests that VvNPR1 is a component of the SA defense signalling pathway in grapevine. This implies the existence of highly conserved mechanisms for regulation of defense gene expression among plant species. As a consequence, overexpression of *VvNPR1 *and other signalling elements has the potential to enhance disease resistance in this crop species. Further work will concentrate on the search for transcription factors interacting with the two VvNPR1 proteins in grapevine, and on the analysis of pathogen tolerance in *npr1 *mutant and wild type Arabidopsis overexpressing *VvNPR1.1 *and *VvNPR1.2*.

## Methods

### Biological material

*Vitis vinifera *cv Chardonnay 96 and *Vitis riparia *cv Gloire de Montpellier were obtained from ENTAV (Etablissement National Technique pour l'Amélioration de la Viticulture, Le Grau du Roi, France). *Vitis vinifera *cv Syrah was provided by INRA (Colmar, France). These clones were propagated on MS medium supplemented with 20 g.L^-1 ^sucrose and 0.7% bacto-agar in a growth chamber at 25°C, under a 16/8 h photoperiod.

Four-week-old *in vitro *plantlets of *Vitis vinifera *cv Chardonnay and *Vitis riparia *were transferred to potting soil (Fertiligène, NFU 44–571) inside a closed translucide propagator under saturating humidity for 7 days. Plantlet acclimatization was realized by gradually raising the propagator's lid. Plants were grown in potting soil for 3 weeks (22°C, 16/8 h photoperiod, 70% humidity) before use for treatments or pathogen inoculation. Eight-week old *in vitro*-grown plants from *Vitis vinifera *cv Syrah were used for *Agrobacterium *infiltration experiments.

*Nicotiana benthamiana *plants were grown in potting soil under a 16/8 h photoperiod for 2 weeks prior to be used for *Agrobacterium *infiltration. *Plasmopara viticola *was kindly provided by Sabine Merdinoglu (INRA, Colmar, France).

### Treatment of plants with chemicals and pathogens

Detached leaves of *Vitis vinifera *cv Chardonnay were sprayed with a BTH solution (80 mg.L^-1^, Bion, Syngenta Agro AG, Dielsdorf, Switzerland). Control leaves were sprayed with water. Leaves were maintained in sealed Petri dishes on humid Whatmann 3 MM paper, collected at different time points and immediately frozen in liquid nitrogen.

For *Plasmopara viticola *inoculation, *Vitis vinifera *cv Chardonnay and *Vitis riparia *plantlets were placed in a closed translucide propagator. Abaxial leaf surfaces were sprayed with freshly collected sporangia propagated on *V. vinifera *cv Chardonnay and resuspended in water at approximately 1.5 × 10^5 ^spores.mL^-1^. Inoculated plants were placed in a growth chamber at 21°C under obscurity for 24 h, then under a 16/8 h photoperiod for 6 days. Inoculation of *Vitis vinifera *cv Syrah was performed by spraying 10^4 ^spores.mL^-1 ^on detached leaves of agroinfiltrated *in vitro-*cultured plantlets that were maintained in sealed Petri dishes on humid Whatmann paper under conditions described above. Leaves were collected at different time points and immediately frozen in liquid nitrogen.

### Cloning of *VvNPR1.1 *and *VvNPR1.2*

The nucleic acid sequence of Arabidopsis *NPR1 *was used to search an EST database of abiotically stressed leaves of *V. vinifera *cv Chardonnay (EST Analysis Pipeline, ESTAP [27], using BLASTN. Two EST with significant similarity to *AtNPR1 *were identified. Full-length cDNA were reconstituted by searching the Genoscope database of grapevine genome sequencing with the two EST previously identified [28]. Full-length cDNAs of *VvNPR1.1 *(GSVIVT00016536001) and *VvNPR1.2 *(GSVIVT00031933001) were amplified from reverse-transcribed RNA from SA-treated Chardonnay leaves using *Pfx *DNA polymerase (Invitrogen). *AtNPR1 *cDNA was amplified from reverse-transcribed cDNA from *Arabidopsis thaliana *Col-0 leaves.

For subcellular localization, the *AtNPR1*, *VvNPR1.1 *and *VvNPR1.2 *coding sequences were cloned by Gateway (Invitrogen) recombination reactions into the pK7FWG2 vector [41], upstream of eGFP.

For transient expression in *N. benthamiana *and grapevine leaves, full-length *AtNPR1*, *VvNPR1.1 *and *VvNPR1.2 *cDNAs were cloned between the CaMV 35S promoter and the 35S terminator sequences of the pUCAP-intron vector [42]. This vector contains an intron between the promoter and the terminator sequence, which was excised and replaced by *NPR1 *cDNA sequences. A six histidine tag coding region was added to the 3' end of each cDNA in order to facilitate detection of transgene product. The cassette containing *AtNPR1*, *VvNPR1.1 *and *VvNPR1.2 *between the CaMV 35S promoter and the 35S terminator was excised by *Asc*I/*Pac*I digestion and cloned into the pBINplus vector [43].

### Sequence alignment and phylogenetic analysis

Protein sequence alignment was realized using the ClustalW program. The phylogenetic tree was constructed with the Phylo_win program [44], using the Neighbor-Joining method. Boostrap values were obtained from 500 replicates.

### Gene expression analysis by semi- quantitative PCR and real-time quantitative PCR

RNA extraction and DNase I treatment were performed as described in Chong et al. [21]. Reverse transcription was performed on 0.5 μg of RNA with the iScript cDNA synthesis kit (Biorad), according to the manufacturer's instructions.

Semi-quantitative RT-PCR was performed by using recombinant Taq DNA polymerase (Invitrogen, Cergy Pontoise, France). Control reactions to normalize RT-PCR were done with primers derived from grapevine actin sequences. PCR on serial dilutions of cDNA were performed at 55°C and 29 cycles to define semi-quantitative conditions that resulted in amplification linear to RNA amounts. The experiments were performed twice with similar results. Primers used for PCR are listed in table 1.

**Table 1:** Sequence of primers used for semi-quantitative RT-PCR in grapevine

Gene	Accession number	Forward Primer 5' → 3'	Reverse Primer 5' → 3'
*VvACT*	AF369524^a^	TGCTATCCTTCGTCTTGACCTTG	GGACTTCTGGACAACGGAATCTC

*VvPR1*	GSVIVT00038575001^b^	GGAGTCCATTAGCACTCCTTTG	CATAATTCTGGGCGTAGGCAG

*VvNPR1.1*	GSVIVT00016536001^b^	GGAATTCGATGTTGGGTACG	GCAACCTTGTCAAGAATGTCC

*VvNPR1.2*	GSVIVT00031933001^b^	GCCGTACGGTAAGGTTGGAT	GAGCCTTCCCGATGAAGTTG

^a ^Genbank accession number

^b ^Genoscope Grape Genome Browser number

For real time PCR, reactions were carried out on the iCycler system (Biorad, Marnes-la-Coquette, France). PCR reactions were carried out in triplicates in a reaction buffer containing 1× iQ SYBR^® ^Green Supermix, 0.2 mM of forward and reverse primers and 10 ng of reverse transcribed RNA in a final volume of 25 μl. Thermal cycling conditions were: 30s at 95°C followed by 40 cycles of 15s at 94°C, 30s at 60°C and 30s at 72°C. Acquisition temperatures were 83°C for *VvPR1 *and 77°C for *VvACT*, *VvSTS*, *VvNPR1*.*1 *and *VvNPR1*.*2*. The calibration curve for each gene was obtained by performing real-time PCR with serial dilutions of the cloned cDNA fragment (from 10^2 ^to 10^8 ^cDNA copy number). The specificity of the individual PCR amplification was checked using a heat dissociation curve from 55 to 95°C following the final cycle of the PCR. The results obtained for each gene of interest at each time point were normalized to the expression of a reference gene (*VvACT1*) and fold induction compared to H_2_O treatment was calculated as described by Pfaffl *et al *[32]. Mean values and standard deviations were obtained from 2 duplicate experiments and are representative of 2 independent experiments. Primers used for real-time quantitative PCR are listed in table 2.

**Table 2:** Sequence of primers used for real-time PCR in grapevine

Gene	Accession number	Forward Primer 5' → 3'	Reverse Primer 5' → 3'
*VvACT*	AF369524^a^	TCCTGTGGACAATGGATGGA	CTTGCATCCCTCAGCACCTT

*VvSTS*	DQ366301^a^	CATCAAGGGTGCTATGCAGGT	TCAGAGCACACCACAAGAACTCG

*VvPR1*	GSVIVT00038575001^b^	GGAGTCCATTAGCACTCCTTTG	CATAATTCTGGGCGTAGGCAG

*VvNPR1.1*	GSVIVT00016536001^b^	GACCACAACCGAGCTTCTTGATCT	ATAATCTTGGGCTCTTTCCGCATT

*VvNPR1.2*	GSVIVT00031933001^b^	GCAGGAAACAAACAAGGACAGGAT	CAGCCATTGTTGGTGAAGAGATTG

^a ^Genbank accession number

^b ^Genoscope Grape Genome Browser number

### Transient expression in tobacco and grapevine leaves

For transient expression in tobacco leaves, we used *Agrobacterium tumefaciens *GV3101 transformed with pK7FWG2 or pBINplus carrying *AtNPR1*, *VvNPR1.1 *or *VvNPR1.2*. An overnight culture of bacteria containing the appropriate construct was resuspended in the same volume of 10 mM MgCl_2_. Bacterial suspension's concentration was adjusted to OD600 = 0.5 with 10 mM MgCl_2_. Acetosyringone (200 μM final) was added to the bacterial suspension prior tobacco leave infiltration using a syringe without needle.

Transient expression in grapevine leave experiments was realized as described in Santos-Rosa *et al*. [34]. *A. tumefaciens *C58CI culture transformed with pBINplus carrying *AtNPR1-His *or *VvNPR1.1-His *was prepared as described [34]. Detached leaves from 8- to 10 week-old grown *V. vinifera *cv Syrah were submerged abaxial face down in cylindrical flasks (40 mL) containing 7 mL of bacterial culture. Leaves were covered by a disk of Miracloth. Flasks were then placed into a dessicator. Vacuum was applied for 2 min at 15 mm Hg with an oil-pump (GmbH, Type N035.3AN.18). Vacuum was applied twice for each leaf. Leaves were then placed in sealed Petri dishes on humid Whatmann paper for three days before harvest or inoculation with *Plasmopara viticola *as described in "Biological materials".

### Subcellular localization of VvNPR1.1 and VvNPR1.2

*AtNPR1*, *VvNPR1.1 *and *VvNPR1.2 *in pK7FWG2 vector [41] were transiently transformed into *Nicotiana benthamiana *by agroinfiltration as described above. Agroinfiltrated leaf sectors were observed 3 days after infiltration. Images were acquired with a LSM510 confocal microscope (Carl Zeiss, software version AIM 4.2), using a 63×, 1.2 NA water immersion objective lens at 23°C. Fluorescence of free GFP or GFP fusion proteins was observed after excitation with a 488 nm laser line, using a 505–550 band-pass emission filter.

### Immunoblot analysis of PR proteins

Foliar explants were harvested from *N. benthamiana *infiltrated with *Agrobacterium *carrying *AtNPR1*, *VvNPR1.1 *and *VvNPR1.2 *in pK7FWG2 vector, 3 days after infiltration.

Total soluble protein was extracted from leaves by grinding in liquid nitrogen and resuspending the powder in extraction buffer as described [30]. The protein concentration of the extract was determined with the Bio-Rad protein assay. SDS PAGE was carried out according to standard procedures with 10 μg of total proteins. Proteins were electro-transfered on Immobilon P membranes (Millipore, Bedford, MA). Detection was realized with the immune-star chemiluminescent kit (Bio-Rad, Hercules, CA). The blots were probed by using polyclonal antisera raised against an acidic PR-1 isoform (PR 1b, [45]), a β-1.3 glucanase isoform (PR-2, [46]) and basic chitinases (PR-3, [47]) purified from tobacco. Polyclonal antisera were kindly provided by M. Legrand (IBMP, Strasbourg, France) and used at a 1:10 000 dilution. Protein loading was checked by Coomassie Blue staining of membranes. The experiment was performed twice with similar results.

## Abbreviations

NPR1: non expressor of *PR *genes 1; SA: salicylic acid; BTH: benzothiadiazole; INA: 2.6-dichloroisonicotinic acid; JA: jasmonic acid; ET: ethylene; PR: pathogenesis related; SAR: systemic acquired resistance; BTB/POZ: broad complex, tramtrack and bric a brac/pox virus and zinc finger; ARD: ankyrin repeat domain; NLS: nuclear localization signal.

## Authors' contributions

GLH carried out most of the experiments, *ie*, gene cloning and phylogenetic analyses, expression studies, transient expression in grapevine, and participated in transient expression in *N. benthamiana*.

TH participated in the design of the study, gave advices for GFP localization experiments and helped to draft the manuscript.

PM participated in the design of the study and helped for transient expression in *V. vinifera*.

JM did the confocal microscopy observations.

BW conceived the study and has done general supervision.

JC carried out transient expression in *N. benthamiana *and western blot analyses, performed conceptual and experimental design and drafted the manuscript.

All authors read and approved the final manuscript.
